# Longitudinal findings of a U.S. preventive evidence-based family intervention tested among youth in Ecuador: Familias Unidas

**DOI:** 10.1371/journal.pgph.0000694

**Published:** 2023-05-25

**Authors:** Yannine Estrada, Alyssa Lozano, Ana M. Quevedo Terán, Daphne G. Eckembrecher, Lourdes M. Rojas, Cecilia Condo Tamayo, Tae Kyoung Lee, María Rosa Velázquez, María I. Tapia, Julio Martin, Guillermo Prado

**Affiliations:** 1 School of Nursing and Health Studies, University of Miami, Miami, FL, United States of America; 2 Department of Public Health Sciences, University of Miami Mill School of Medicine, Miami, FL, United States of America; 3 Universidad Católica de Santiago de Guayaquil, Guayaquil, Ecuador; 4 School of Education and Human Development, University of Miami, Miami, FL, United States of America; PLOS: Public Library of Science, UNITED STATES

## Abstract

Interventions that address adolescent conduct problems are essential for decreasing negative risk behaviors and promoting positive protective factors among youth. Although interventions have been developed and tested in the United States, preventive evidence-based interventions (EBIs) are less available in Latin American countries such as Ecuador. Therefore, the purpose of this study was to evaluate the efficacy of an evidence-based, parent-centered intervention, *Familias Unidas*, in preventing/reducing conduct problems, across time, among youth in Guayaquil, Ecuador. Ecuadorian youth (ages 12 through 14) and their respective primary caregiver were recruited from two public schools and randomized to either Familias Unidas or Community Practice. A series of latent growth models were run to test for differences between Familias Unidas and Community Practice on conduct disorder symptoms across three timepoints covering 6 months. Ecuadorian mental health professionals were trained to deliver the evidence-based intervention. Findings indicate no direct relationship between condition and average change in conduct problems at 6 months post baseline. However, indirect effects favoring Familias Unidas over Community Practice were found through improvements in family functioning. Findings highlight that Familias Unidas was efficacious in an international setting and indicate the viability of successfully delivering preventive EBIs in Ecuador.

## Introduction

Youth with conduct problems are at higher risk for engaging in sexual risk taking [1–3] and are more likely to abuse substances [4]. Conduct problems are externalizing behaviors that are characterized by persistent patterns of antisocial behaviors that violate age-appropriate social norms [5,6]. These behaviors may include aggression to people and/or animals, destruction of property, and/or serious violations of rules [7]. Family-based preventive interventions have been efficacious in the prevention of adolescent behavioral concerns [8,9]. by targeting common underlying risk and protective factors such as parent-adolescent communication [10,11]. For example, one family-based preventive intervention that has been efficacious and effective in the reduction and/or prevention of adolescent health problems by targeting family-related variables is *Familias Unidas* [12]. Familias Unidas places parent(s) as the agent of change through family-centered, multi-parent groups. Parenting skills are discussed and role-played in eight group sessions and are then applied with the parent and the adolescent in the four family sessions. Parent group sessions focus on parental investment in the adolescent’s peer and school worlds, family communication, family support, positive parenting, parental monitoring, adolescent substance use, and adolescent unsafe sex. During the group sessions, two facilitators offer support for parents and in the family sessions, facilitators assist families in practicing skills and restructuring family interactions. In the United States, Familias Unidas has been implemented in school [13], juvenile justice [11], and primary care [14] settings. In three randomized clinical trials [13,15,16] with Hispanic youth [ages 13–17], Familias Unidas was efficacious/effective in preventing and reducing drug use, and sexual risk behaviors and effects have been partially explained by improvements in family protective factors (e.g., parent-adolescent communication).

While family-based prevention interventions such as Familias Unidas have been developed and tested in the U.S. and high-income countries [17], it is unclear whether these prevention interventions maintain their efficacy when transposed to a different context from the one in which they were originally developed and tested (i.e., in low-and-middle income countries) [18,19]. In a recent review of family skills training programs to prevent alcohol and drug use in Latin America [20], only *Familias Fuertes* [21] had been evaluated in Ecuador. However, this evaluation was not a randomized clinical trial design, thereby limiting our ability to make inferences on its efficacy in preventing and/or reducing alcohol and drug use [6,20]. There have been family skills training programs developed within other Latin American reigions, for example, *ConSentido* (With Sense) in Colombia [22] and *Prevenir en Familia* (Prevent in the Family) in Chile [23]. However, despite their availability locally, these programs lack rigorous evidence to suggest that they prevent alcohol and drug use [20]. The lack of efficacy data supporting interventions is concerning given that behavioral disorders pose serious health concerns among Hispanic adolescents globally. Further, conduct problems such as youth violence and aggression are comorbid and present a global disease burden among youth [24]. Specifically, conduct problems are associated with a wide range of behavioral and psychosocial outcomes [25] such as early onset of substance use [26] and early sexual behavior [2].

To increase preventive interventions for the above-mentioned behavioral concerns, researchers at the Universidad Católica de Santiago de Guayaquil (UCSG; Catholic University of Santiago of Guayaquil) collaborated with the University of Miami (UM) to deliver Familias Unidas in the U.S. to prevent conduct problems. The UCSG selected to test Familias Unidas due to its Hispanic culturally centered approach, emphasis on improving family functioning, and evidence base in reducing conduct problems [18]. Furthermore, the intervention’s underlying framework, ecodevelopmental theory [27], focuses on targeting risk and protective factors at multiple levels of the adolescent’s eco-system (e.g., family, peers, school), which is well aligned with the goals of researchers in Ecuador.

It is unknown whether Familias Unidas works similarly in Hispanic populations outside of the United States. The current manuscript seeks to examine the six-month long-term effects of Familias Unidas on conduct problems among families in Guayaquil, Ecuador. An article on the immediate post-intervention effects has been published [6]. We hypothesized that, across time, Familias Unidas would be more efficacious than Community Practice in reducing conduct problems. Additionally, given that family functioning has, in past Familias Unidas trials, served as a mediator between condition and main outcomes (e.g., [28]), we hypothesized that intervention effects would be partially mediated by family functioning.

## Methods

### Ethics statement

This two-armed randomized controlled trial was approved by the Ethical Committee of the General Hospital Luis Vernaza, the Ministry of Public Health in Ecuador, and the University of Miami Institutional Review Board. Deviations from the study protocol were addressed and documented. Written consent was obtained from parents/guardians. Formal consent was obtained from parents/guardians for child participation. Written assent was also obtained for child participants.

### Inclusivity in global research

Additional information regarding the ethical, cultural, and scientific considerations specific to inclusivity in global research is included in the Supporting Information (S1 Checklist).

### Procedures

**Fig 1:**
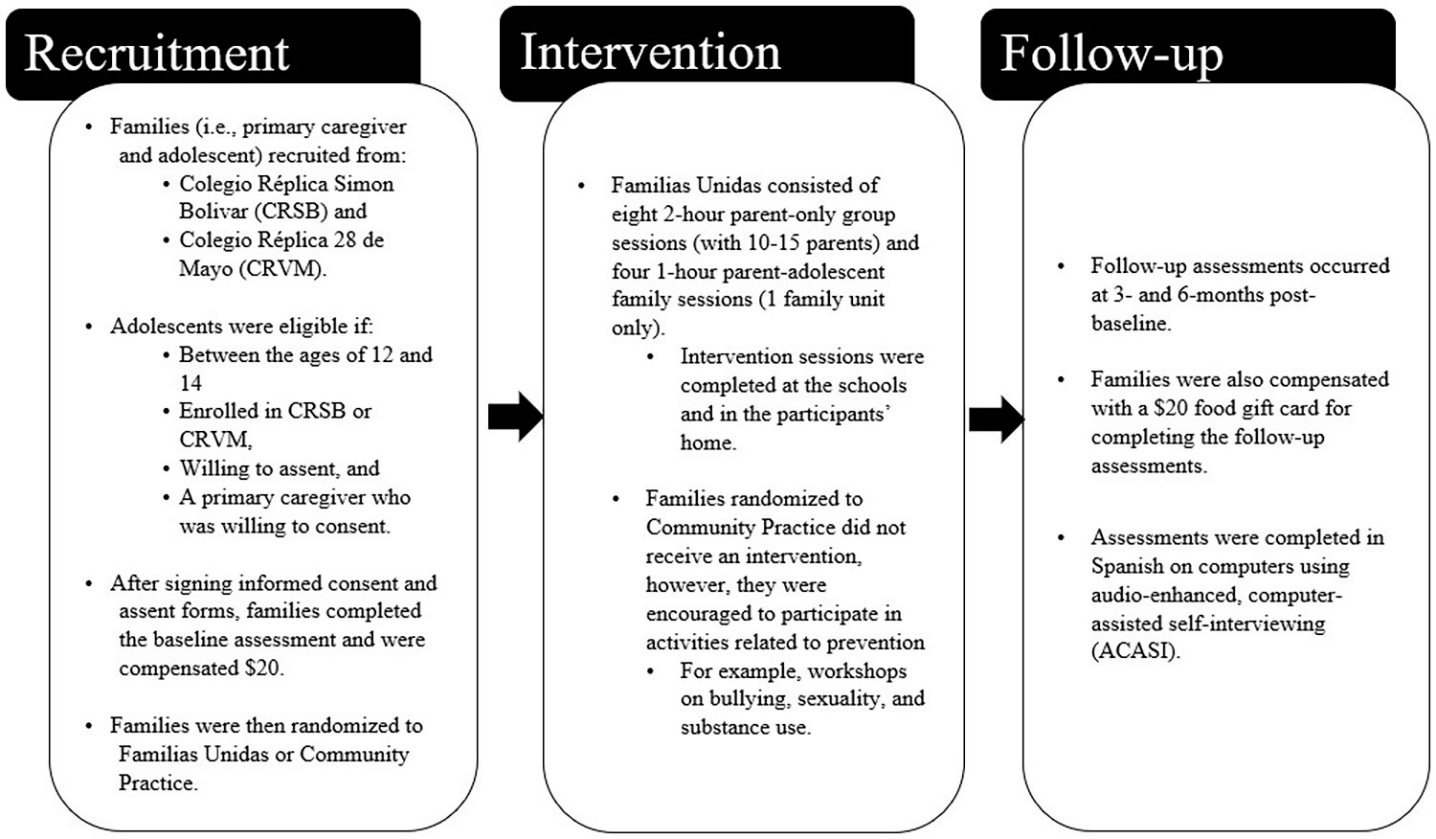
Recruitment, intervention, and follow-up process.

Recruitment, intervention, and follow-up assessments took place from May 2015 through February 2016 and are described below (see Fig 1).

#### Recruitment

Families, defined as an adolescent and a primary caregiver (i.e., mother, father, or legal guardian), were recruited from two public schools: Colegio Réplica Simon Bolivar (CRSB) and Colegio Réplica 28 de Mayo (CRVM). These schools are in a low-income, urban setting and were selected because of their location within a district without access to prevention programs not provided by the ministry of education. The Ecuadorian investigators wanted to select two schools within a particularly marginalized community. School wide screenings and referrals from school personnel were utilized to recruit adolescents who met the study’s eligibility criteria: adolescents between the ages of 12 and 14, enrolled in CRSB or CRVM, willing to assent, and a primary caregiver who was willing to consent. Screenings were conducted through parent and student gatherings held at the school. Families were only excluded if they had a prior psychiatric hospitalization. Across the two schools, recruiters screened 248 adolescents and their primary caregivers, as described in the published CONSORT diagram [6]. Informed consent was written.

After the primary caregiver and adolescent signed the informed consent and assent forms, respectively, they completed the baseline assessment. Families in both conditions were compensated with a $20 food gift card for completing the baseline assessment (Ecuador utilizes the U.S. dollar). After baseline assessments, sealed envelopes were used to execute the randomization of study conditions. Randomization was at the individual level within two schools, stratification was not done at the school level. Randomization resulted in 129 families in Familias Unidas and 110 in Community Practice. Research personnel were not blinded to the study condition assignment. Assessment data were collected at the adolescents’ school.

#### Intervention study conditions

Familias Unidas consisted of eight 2-hour parent-only group sessions (with 10–15 parents) and four 1-hour parent-adolescent family sessions (1 family unit only) led by a UM trained facilitator from the UCSG. Intervention sessions were completed at the schools and in the participants’ home. A detailed description of the intervention is available in the literature [12]. Details on the program adaptation can be found elsewhere [18], however, briefly, during the initial feasibility study of the Familias Unidas intervention in Ecuador, the UCSG only made minor adjustments in wording and updated prevalence rates of drug use and risky sexual behavior to reflect Ecuadorian population. There were no deep level structural changes that modified program components or structure [18]. Trained facilitators who were interventionists and students in their last year of social work at the UCSG delivered the 12-week using an intervention manual containing all the content to be delivered. Families randomized to the intervention condition were compensated $2 for transportation to attend the group sessions. The sessions were sequenced such that parenting skills were discussed and role-played during the parent group session, and then applied and practiced with the adolescent in the family sessions. Each session built upon one another as the parents were empowered to set goals, communicate with their adolescents, monitor their adolescent’s peers, and practice prevention in everyday life.

*Facilitator training and supervision*. Familias Unidas facilitators received four days of training which consisted of: Understanding the theory behind Familias Unidas, learning the research study protocol (e.g., consent, assessment, randomization), practicing how to engage and retain families, and observing/role playing group and family sessions to better follow the content in the Familias Unidas manuals. Additionally, facilitators were trained on the foundational aspects of research and clinical trials. Throughout the study, the UCSG clinical supervisor delivered two hours of weekly supervision to provide clinical feedback to the facilitators.

*Community Practice*. Families randomized to the control condition did not receive an intervention from the study staff. The community had several workshops and group activities that covered similar prevention topics; however, we did not measure dosage. Participants were verbally encouraged to participate in these activities. Activities related to prevention included workshops on topics such as bullying, sexuality, and substance use. Additionally, there were activities in the local church that involved youth groups.

#### Follow-up assessments

The study consisted of two follow-up time points: 3- and 6-months post-baseline. Families in both conditions were also compensated with a $20 food gift card for completing the follow-up assessments. Assessments were completed in Spanish on computers using audio-enhanced, computer-assisted self-interviewing (ACASI) [29].

### Participants

Table 1 depicts demographic information about the sample of 239 parent-adolescent dyads. Participants consisted of adolescents with a mean age of 12.87 years (*SD* = 0.77). Adolescents were 51.7% female, whereas parents were 93.7% female. The mean parent age was 37 years old (*SD* = 6.75). Forty percent of parents reported being married while 46% reported being in a domestic partnership.

**Table 1:** Baseline comparisons of demographic characteristics by intervention condition.

Variable	Familias Unidas (n = 129)		Community Practice (n = 110)		
	n (%)	Mean (SD)	n (%)	Mean (SD)	*t-* or χ^2^ value (*p*-value)
Relationship to Adolescent					
*Mother*	120 (93.0)		104 (94.5)		1.44 (.49)
*Father*	5 (3.9)		5 (4.5)		
*Legal Guardian*	4 (3.1)		1 (0.9)		
Mean age (Parents)		36.84 (6.74)		37.57 (6.57)	0.83 (.41)
Mean age (Adolescents)		12.85 (0.77)		12.88 (0.73)	-0.29 (.78)
Marital status					
*Married*	39 (35.5)		42 (32.6)		1.00 (.96)
*Widow/Widower*	2 (1.8)		4 (3.1)		
*Single*	13 (11.8)		16 (12.4)		
*Divorced*	5 (5.4)		4 (3.1)		
*Liberal Union*	43 (39.1)		54 (41.9)		
*Separated*	8 (7.3)		9 (7.0)		
School District					
*CRSB*	73 (57.0)		62 (56.4)		
*CRVM*	56 (43.0)		48 (43.6)		
PAC (at baseline)		72.30 (8.48)		71.43 (8.43)	0.59 (.43)
POS (at baseline)		19.61 (4.20)		19.18 (4.01)	0.63 (.42)
PER (at baseline)		9.06 (4.52)		9.17 (3.98)	0.40 (.84)
Conduct problems (at baseline)		6.82 (2.50)		7.92 (2.68)	1.07 (.30)

*Note*. SD = Standard Deviation. *t*-tests were conducted for mean comparisons. Chi-square were conducted for % comparisons. PAC = Parent-Adolescent Communication. POS = Positive Parenting. PER = Parental Monitoring of Peer. CRSB = Colegio Replica Simon Bolivar, CRVM = Colegio Replica 28 de Mayo.

### Measures

To examine the longitudinal effects of Familias Unidas, measures included demographics; a latent family functioning construct consisting of parent-adolescent communication, parental monitoring of peers, and positive parenting; and adolescent conduct problems. These measures were slightly modified (e.g., word choice, phrasing) after feedback from participants in consultation with both UM and UCSG [18].

#### Demographics

Demographic information included gender, age, and marital status for parents. Adolescents were asked about their age and grade.

#### Main outcomes

Adolescent conduct problems were assessed from the parent perspective with the conduct disorder scale from the *Revised Behavior Problem Checklist* (22 items, Cronbach’s α = .94) [30]. The RBPC is a commonly used measure with established reliabilities [30]. Parents rated 22 behavior problems that their adolescent may exhibit on a range from 0–2 (0 = *No problem*, 1 = *Mild problem*, 2 = *Severe problem*). Sample behaviors included stealing from outside the home and inability to work independently.

#### Secondary outcome—Proposed mediator

Family functioning was assessed with parent reports of three indicators: (1) parent-adolescent communication, (2) parental monitoring of peers, and (3) positive parenting. The *Parent-Adolescent Communication Scale* (PAC, 20 items, Cronbach’s α = .73) [31] was used to assess parental report of family communication. The PAC assesses the degree of open communication between parent and child. Parents responded to items utilizing a 5-point Likert scale ranging from 1 = *Strongly disagree* to 5 = *Strongly agree*. The *Parent Relationship with Peer Group Scale* (5 items, Cronbach’s α = .55) [32] was used to assess parental monitoring of peers. This measure assesses how well parents know the adolescent’s friends. Items were rated on a scale from 0 = *Not at all* to 4 = *Extremely often*. The *Parenting Practices Scale* (9 items, Cronbach’s α = .76) [33] was used to assess positive parenting which assesses positive reinforcement for adolescent behaviors. Items were rated on a scale from 0 = *Never* to 4 = *Always*. A single family-functioning variable was used by creating a latent construct consisting of the three indicators described above.

#### Intervention fidelity

The UCSG staff conducted intervention fidelity ratings via observations of recorded parent group and family sessions. Session quality was rated on a 0 = *Not at all/very poor* to 6 = *Extensively/excellent* scale. To assess inter-rater reliability, UM rated 20% of UCSG’s rated group sessions and 10% of UCSG’s rated family sessions.

#### Data analytic strategy

Data analyses for this study consisted of four steps. First, we tested for significant differences in attrition rates with Chi-square tests. Next, to examine potential baseline differences in demographics and the outcome variables by condition, we used Mann-Whitney U Tests (for count variables), independent t-tests (for continuous and normally distributed variables), and Chi-square tests (for categorical variables). Third, to examine the direct effects of Familias Unidas on longitudinal changes in the main outcome (i.e., conduct problems), we used latent growth modeling [34]. Time scores for the growth models were centered at the first time point (i.e., time coding of baseline = 0), across all growth models. The slope trajectory was estimated after controlling for the effects of the intercept by regressing the slope trajectory on initial levels. Therefore, the adjusted trajectory of the targeted outcome was estimated accounting for the baseline variation in the outcome [35]. Next, we tested for intervention effects on the family functioning latent variable, and its indicators, from baseline to three months post-baseline with structural equation modeling (SEM), controlling for baseline levels of the latent family functioning variable. Due to measurement of the latent family functioning variable at baseline and 3-months post-baseline, we conducted a longitudinal measurement invariance test [36] to determine if the same latent family functioning variable was assessed across the two time points. If there was a significant intervention effect on the latent family functioning variable, as a post-hoc test, the latent family functioning variable was decomposed to examine indirect intervention effects on each of the family functioning indicators. Finally, we used the product of coefficients method to test whether the family functioning indicators mediated the effects of study condition on the outcome [37].

In relation to fit indices, the comparative fit index (CFI) and the Tucker–Lewis index (TLI) were used to determine model fit. Values greater than .95 and .90 for CFI and TLI, respectively, typically indicate excellent and acceptable fits to the data [38]. Standardized path coefficients (*r*) are reported as effect sizes [39]. We used full information maximum likelihood (FIML) to address missing data for the repeated measures. To obtain accurate standard error estimates, a complex survey design was used to adjust for the clustering effect of students within schools. All analyses were conducted using M*plus* version 7.2 [40].

## Results

### Comparability of conditions at baseline and inter-rater reliability

There were no significant differences at baseline, by condition, on any of the demographic characteristics, family functioning, or the adolescent main outcome (Table 1). The average session quality was 4.5 (*SD* = .91) out of 6. Kappa scores were acceptable (*k* = .87).

### Main outcomes: Tests of intervention effects

Results showed that overtime, the trajectory of conduct problems decreased for participants in both Familias Unidas (slope = -0.50, 95% CI = -0.824, -0.172, *p* < 0.01) and in Community Practice (slope = -0.44, 95% CI = -0.991, 0.117, *p* = 0.122), but the trajectory was not significant for Community Practice. Intervention condition did not directly predict conduct problem trajectories (standardized beta = -0.11, 95% CI = -0.304, 0.067, *p* = 0.173; CFI / TLI = 0.96 / 0.93). Longitudinal measurement invariance for the latent family functioning variable was tested via comparison of three nested models: (a) configure invariance (unconstrained model), (b) metric invariance in which the corresponding factor loadings were equivalent across measurement (i.e., time points), and (c) scalar invariance, in which the respective factor loadings and indicator variable intercepts were equivalent across time. Next, the nested models were evaluated by comparing the comparative fit index (CFI; ≤ 0.01 invariance holds) [41].

**Fig 2:**
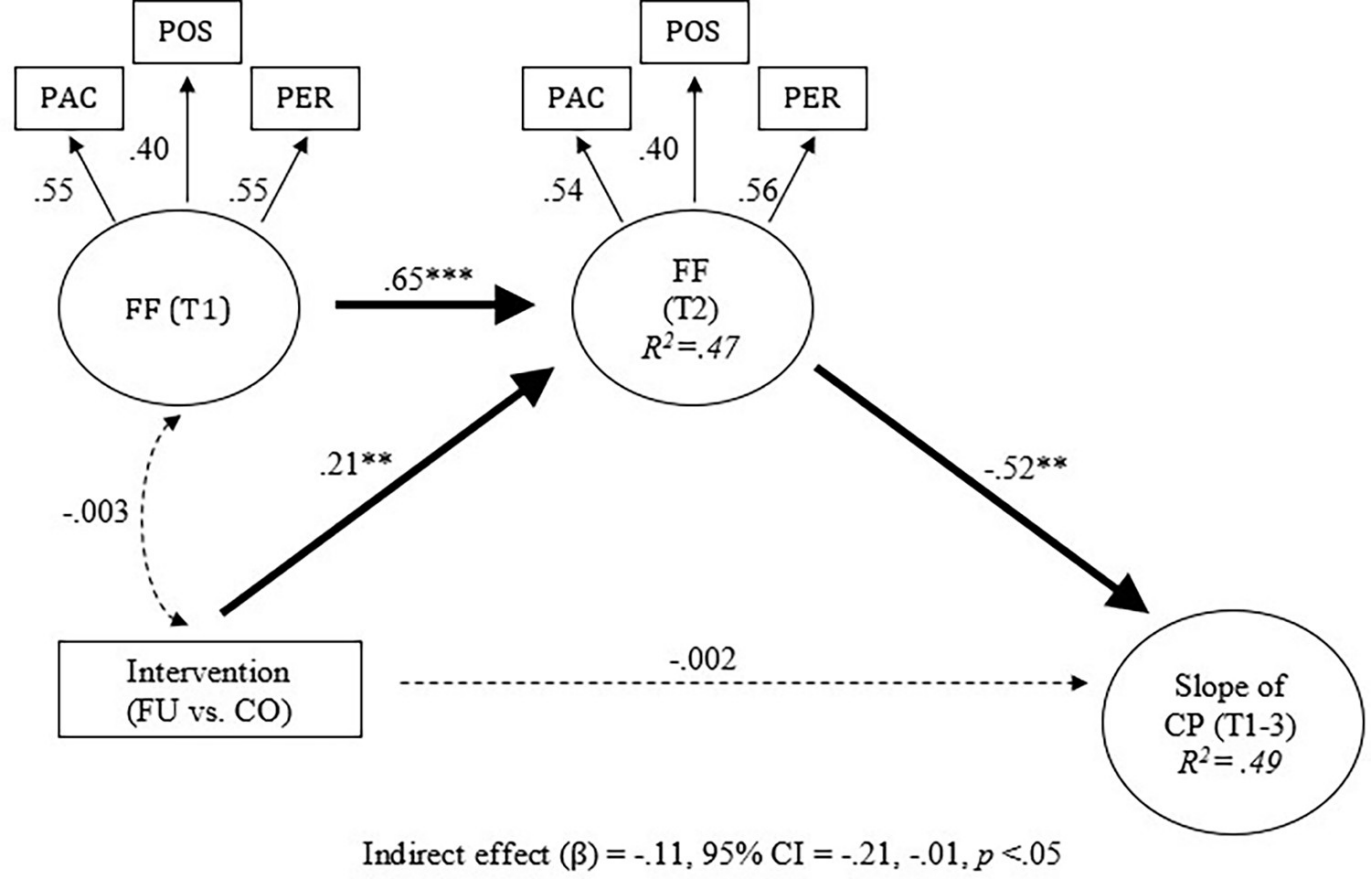
Indirect effects of intervention on trajectories of conduct problems. Note. All coefficients were standardized. All factor loadings were significant at *p* < .001. T = Time point. FU = Familias Unidas. CO = Community Practice. PAC = Parent-Adolescent Communication. POS = Positive parenting. PER = Parental monitoring of Peer. FF = Family function. CP = Conduct problem. Dotted line represented non-significant paths. Initial level effects of conduct problems on the slope growth factor was adjusted (standardized β = -.47, p < .05). X^2^(df) = 57.02 (31). *p* < .01. CFI / TLI = .95 / .93. **p < .01. ***p < .001.

**Fig 3:**
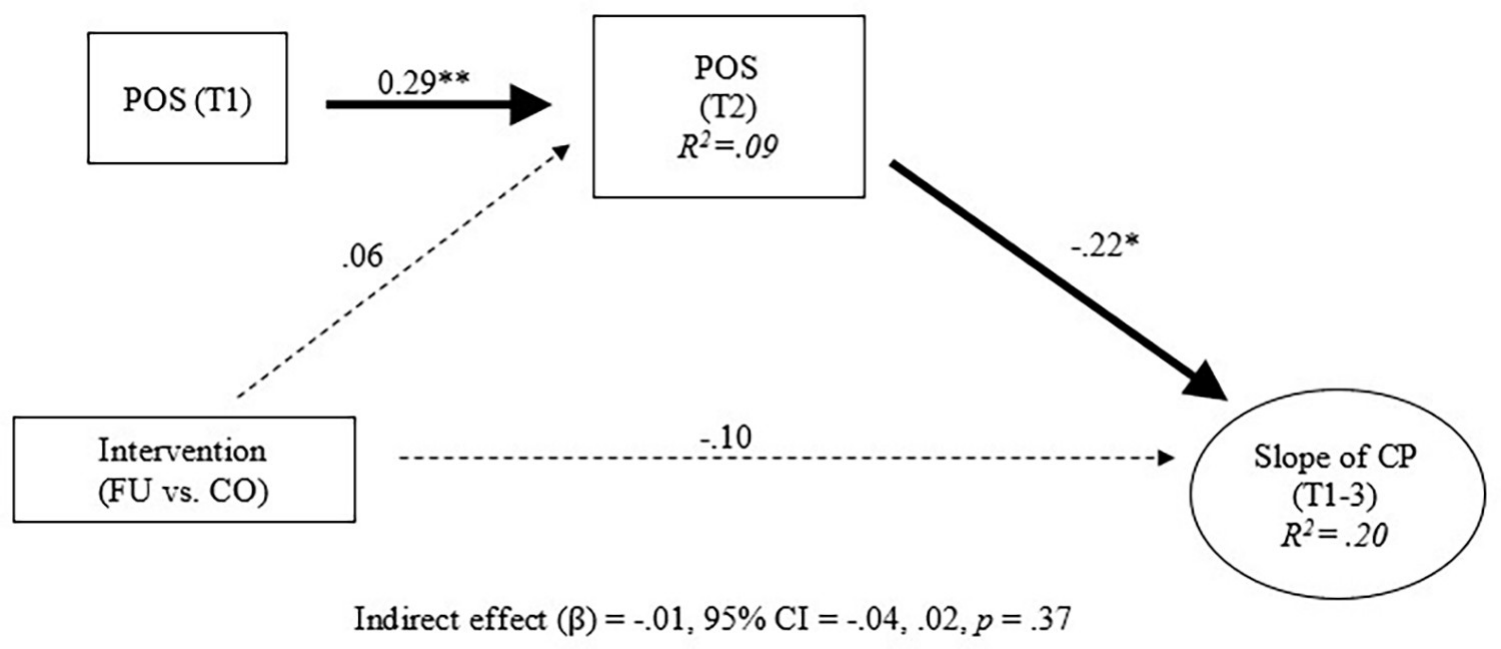
Indirect effects of intervention on trajectories of conduct problems: Parent-adolescent communication. Note. All coefficients were standardized. T = Time point. FU = Familias Unidas. CO = Community Practice. PAC = Parent-Adolescent Communication. POS = Positive parenting. PER = Parental monitoring of Peer. FF = Family function. CP = Conduct problem. Dotted line represented non-significant paths. * *p* < .05. **p < .01. ***p < .001.

**Fig 4:**
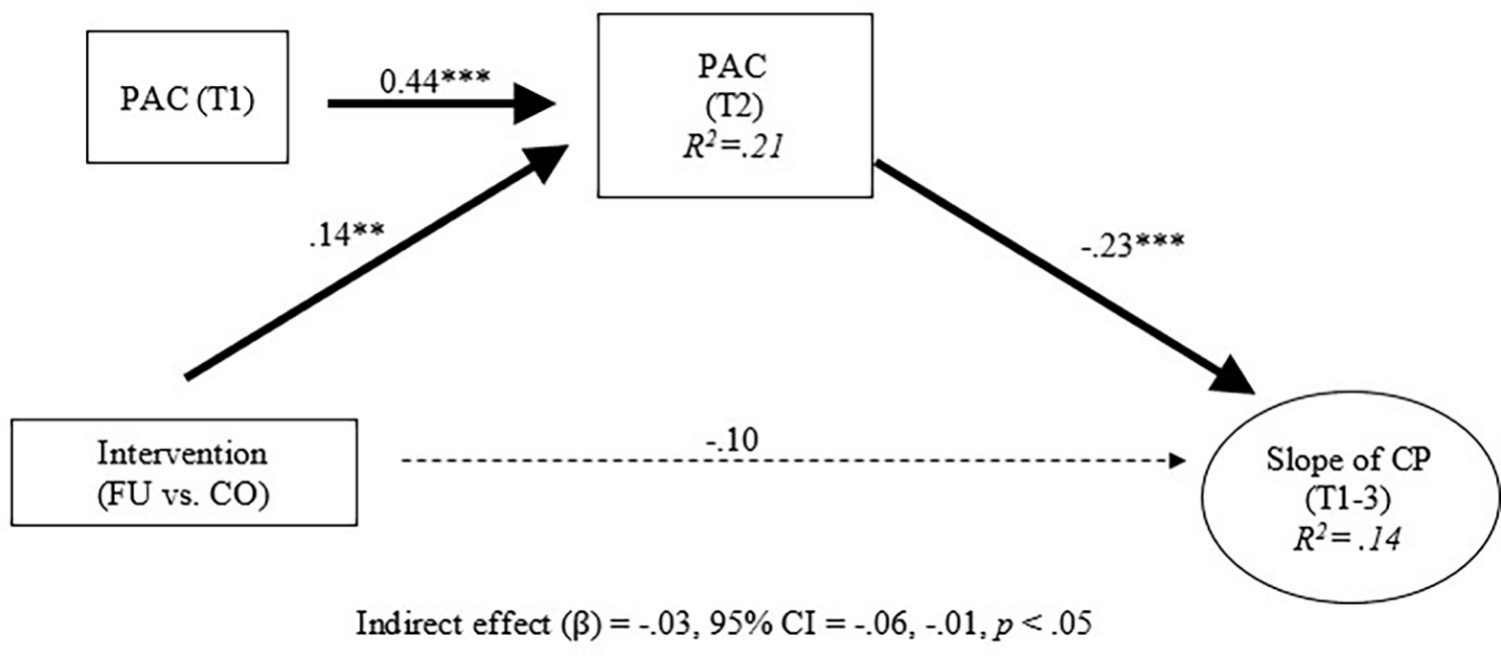
Indirect effects of intervention on trajectories of conduct problems: Parental monitoring of peers. Note. All coefficients were standardized. T = Time point. FU = Familias Unidas. CO = Community Practice. PAC = Parent-Adolescent Communication. POS = Positive parenting. PER = Parental monitoring of Peer. FF = Family function. CP = Conduct problem. Dotted line represented non-significant paths. * *p* < .05. **p < .01. ***p < .001.

**Fig 5:**
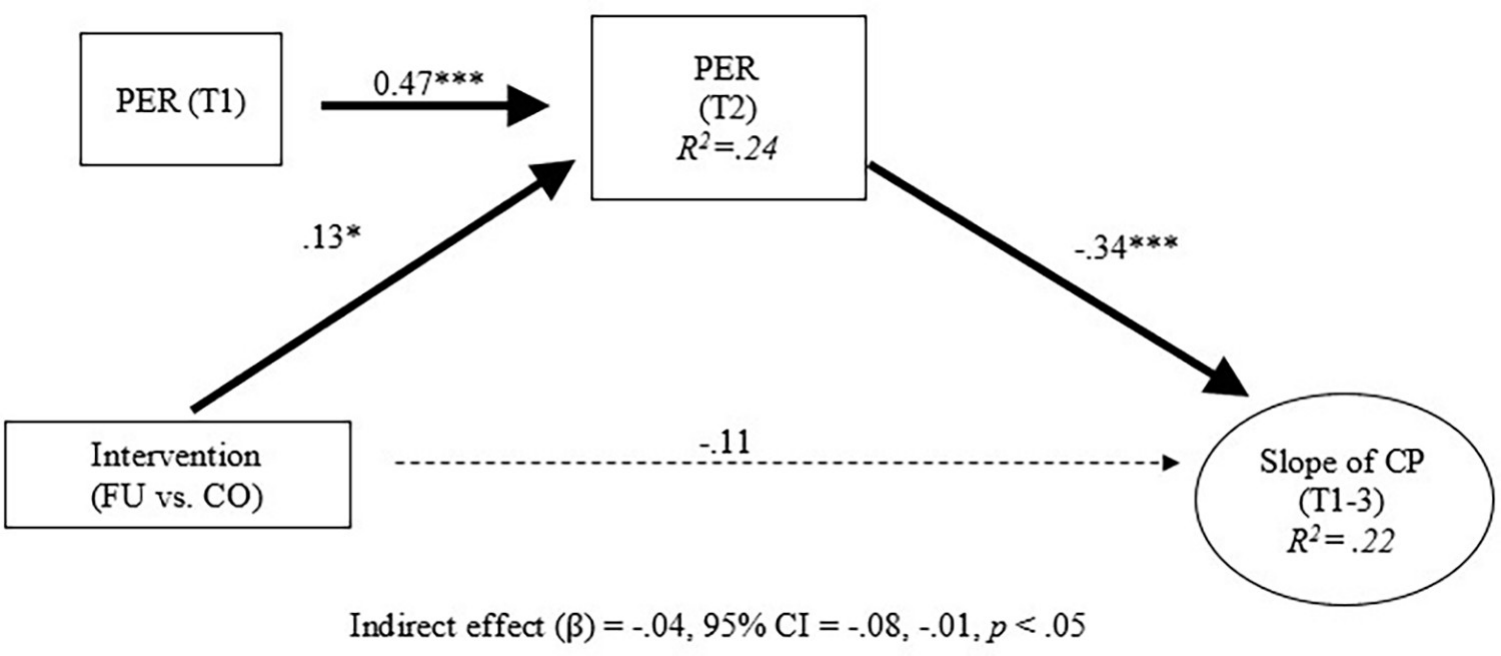
Indirect effects of intervention on trajectories of conduct problems: Positive parenting. Note. All coefficients were standardized. T = Time point. FU = Familias Unidas. CO = Community Practice. PAC = Parent-Adolescent Communication. POS = Positive parenting. PER = Parental monitoring of Peer. FF = Family function. CP = Conduct problem. Dotted line represented non-significant paths. * *p* < .05. **p < .01. ***p < .001.

Intervention and conduct problem trajectories were then added into the longitudinal invariance model. Next, indirect intervention effects were examined. The model fit was acceptable (CFI / TLI = 0.95 / 0.93). Results indicate that, after adjusting for the effects of family functioning at baseline, the intervention increased family functioning at 3-months post-baseline (Fig 2; standardized beta = 0.21, 95% CI = 0.070, 0.352, *p* < 0.01). In addition, family functioning at 3-months post-baseline reduced conduct problems at 6-months post-baseline (Fig 2; standardized beta = -0.52, 95% CI = -0.88, -0.18, *p* < 0.001). This mediation effect was statistically significant (standardized beta = -0.11, 95% CI = -0.21, -0.01, *p* < 0.05). Post hoc analyses were conducted to determine indirect effects for each of the family functioning indicators proposed in this study. Therefore, the family functioning construct was decomposed into each of its indicators (i.e., parent-adolescent communication, parental monitoring of peers, and positive parenting) to test for indirect intervention effects (Figs 3–5). Results showed significant indirect intervention effects on two of the three individual family functioning indicators after adjusting for the effects of these indicators at baseline (parent-adolescent communication: standardized beta = -0.03, 95% CI = -0.06, -0.01, *p* < 0.05, Fig 3; parental monitoring of peers: standardized beta = -0.04, 95% CI = -0.08, -0.01, *p* < 0.05, Fig 4). However, no significant indirect intervention effects were shown for positive parenting (standardized beta = -0.01, 95% CI = -0.04, 0.02, *p* = 0.37, Fig 5).

## Discussion

The present study evaluated the effects of a prevention intervention on conduct problems (e.g., disruptive behavior, argumentative, impertinent) among a sample of Ecuadorian youth. Findings showed that, compared to Community Practice, the Familias Unidas intervention did not directly impact conduct problem trajectories across three time points. The nonsignificant findings may be related to the short 6-month follow-up period; past trials have found effects on adolescent behavioral problems at later timepoints (i.e., 12-, 18- or 24-months post baseline). Nonetheless, Familias Unidas was efficacious in improving family functioning three months post baseline, which aligns with completion of the intervention. Further, family functioning three months after baseline predicted adolescent conduct problems at six months. Hence, Familias Unidas intervention effects were indirect, reducing adolescent conduct problems by first impacting family functioning.

While some may argue that there are no differences between U.S. Hispanic populations and those abroad, we cannot assume that interventions developed and tested in the U.S. can be transposed to other Hispanic contexts. Indeed, in our preparatory work before this study, it was necessary to make modifications to the intervention and the wording of the assessment battery [18]. For example, words that are commonly used in Miami, Florida were not understood or had a different meaning in Ecuador.

Beyond the gains of the families who participated in the intervention condition, the collaboration between UM and the UCSG involved training in the conduct of research to equip UCSG with the tools needed for continued evaluation of Familias Unidas and any future prevention interventions of interest. To move the prevention field forward, it is crucial to capacitate systems in the conduct of research and rigorous execution of protocols to implement efficacious, evidence-based programming to populations that most need them. However, training unequipped institutions in rigorous research methodology is an often-overlooked component in the dissemination of evidence-based practice, although it is one that is needed for the successful translation of interventions and promotion of youth health [42]. Oftentimes, these organizations lack the infrastructure to conduct research. Further, implementation of prevention interventions in different settings from which they were tested involves careful attention to local differences in language and local cultures, even within the same ethnic population, particularly if the adaptation occurs across international lines.

There are several limitations to the current study. First, the sample came from only two schools in Guayaquil, therefore, findings are not generalizable to other populations. For example, given the low socio-economic status of our sample, findings may not generalize to other Ecuadorian sub-populations such as less marginalized ones. Second, the possibility exists that, like most research with human subjects, there was selection bias as parents involved in the study may have been more motivated to participate. Future research should consider gathering information on participants who decline participation to determine if there are any differences between families who engage/participate, and those who do not. Third, all measures were self-reported, and therefore it is possible that participants responded in a pro-social manner. Relatedly, we were only able to collect three time points, of which the third time point was 6 months post baseline (i.e., 3 months post intervention completion). Since previous trials have found effects at 12-, 18- or 24-months post baseline, this may limit our findings on the efficacy of the intervention on adolescent behavioral problems over time. Lastly, we did not assess the dosage of the families in the community practice condition, thereby limiting our ability to assess how the workshops and group activities in that condition compared to the Familias Unidas intervention.

In summary, the current study demonstrates the efficacy of Familias Unidas to reduce conduct problems in Ecuadorian adolescents through improvements in family functioning. Other family-based programs evaluated outside the U.S. (i.e., in Latin America) may not use rigorous randomized controlled trial designs such as the one employed in this study, thereby prohibiting researchers from making inferences on the efficacy of those programs. These findings highlight how evidence-based interventions in the U.S. could also prove efficacious in countries such as Ecuador, where there is a critical need for prevention and emerging infrastructure to test and sustain interventions.

## Supporting information

S1 Checklist Inclusivity in global research. (DOCX)

S1 Dataset Study data set. (CSV)
